# The prevalence of sickness absence among primary school pupils – reason to be worried?

**DOI:** 10.1186/s12889-021-10193-1

**Published:** 2021-01-20

**Authors:** Esther Karen Pijl, Yvonne Theodora Maria Vanneste, Angelique Eveline de Rijk, Frans Joseph Maria Feron, Jolanda Mathijssen

**Affiliations:** 1 Child and Youth Healthcare Department, GGD West-Brabant, Breda, The Netherlands; 2 Department of Social Medicine, Faculty of Health Medicine and Life Sciences, Care and Public Health Research Institute (CAPHRI), Maastricht University, Maastricht, The Netherlands; 3 Dutch Knowledge Centre for Youth Health (NCJ), Utrecht, The Netherlands; 4 Academic Collaborative Centre Youth, Tranzo, Tilburg University, Tilburg, The Netherlands

**Keywords:** Primary education, Primary school pupils, School absenteeism, Sickness absence

## Abstract

**Background:**

Absence from school can lead to lower educational achievement and poor health. Little is known about school absence in primary education. This study’s first aim was to examine the prevalence of school absence in primary schools and differing types of absence, including sickness absence. The second aim was to determine which pupil characteristics and types of absence were associated with extensive sickness absence.

**Methods:**

The school absence registries for the school year 2015–2016 were analysed retrospectively in eight mainstream primary schools with 2216 pupils, and six schools for special primary education with 1000 pupils in the West-Brabant region of the Netherlands. Descriptive analyses, χ^2^-tests, Mann-Whitney U tests and logistic regression analyses were performed.

**Results:**

The one-year prevalence of school absence was 85% in mainstream primary schools and 79% in special schools. Sickness absence was the most prevalent type of absence, occurring in 75 and 71% of pupils, respectively The prevalence of extensive sickness absence was 13 and 23%, respectively. In mainstream schools, extensive sickness absence was associated with a young age, low parental educational level, more doctor’s visits and unauthorised absence, and in special schools with more doctor’s visits, other authorised absence, tardiness and unauthorised absence.

**Conclusions:**

The prevalence of extensive sickness absence was high, and as this was associated with other types of absence, these pupils missed even more days of school. Public health research, policy and practice should address sickness absence among primary school pupils, to prevent adverse effects on children’s development.

## Background

Absence from school is an important public health issue as it can lead to lower educational achievement, social difficulties, risk behaviour, school dropout, and ultimately, poor health [1–8]. School absence can be caused by underlying problems of both medical and social origin. It has been associated with chronic illness, psychiatric problems, bullying, child abuse, poverty, low parental educational levels and school-related problems [9–20]. Although research often focuses on secondary education, absence from school in primary education impacts educational achievement negatively and the habit of missing school can start during primary education [7, 8, 10, 21–23].

The prevalence of school absenteeism in primary education is unclear. Previous studies have examined varying types of absence (often unauthorised absence or truancy), and have used varying subpopulations (e.g. chronically ill pupils), differing methods of measurement (e.g. mean or median days or absence rates), and differing thresholds to define problematic absence. Depending on the threshold used (varying from 2 to 20 days), reported percentages of problematic absence in primary education lie between 3 and 48% [10, 24, 25].

This study’s first aim is to explore the prevalence of school absence in regular primary education. This exploration was done in the Netherlands where children attend either regular primary education (approximately 95% of children) or special needs education [26]. There are two types of regular primary education: a mainstream primary school (MPS), and a special school for primary education (SSPE). The latter provides additional support for mild learning difficulties, behavioural problems and parenting problems. Special needs education is for pupils with chronic illness, disabilities or severe learning and behavioural problems. Both MPS and SSPE were included in this study, special needs education schools were not.

Dutch legislation differentiates between unauthorised absence (e.g. truancy) and authorised absence (e.g. absence due to sickness). In the Netherlands, unauthorised absence is overseen by school attendance officers who can use penalties to enforce the law. However, sickness absence is not addressed systematically [27], even though this is the most common type of absence in Dutch secondary education [28]. The situation is hypothesized to be similar in primary education. Pupils who are extensively reported sick (more than nine days or more than four periods in a school year) [29] are likely to be at risk of the negative consequences of absence, as they miss a substantial number of lessons and peer contact. It is important to identify these pupils, so this study’s second aim is to gain insight into the characteristics of pupils who are extensively absent due to sickness.

This study examines two research questions.

(i) What is the prevalence of school absenteeism in regular primary education?
(ii) How are pupil characteristics and other types of absence related to extensive sickness absence?

## Methods

### Primary schools

The schools included in the present study were participating in a research project exploring school absence in primary education in the West-Brabant region of the Netherlands. The number of schools approached was based on a power analysis carried out for another study in that research project, for which 10 MPSs were needed.

Regular education schools were included in this study. Special needs schools were excluded as these are intended for pupils with severe physical or psychiatric problems [30], which could seriously influence attendance patterns. A random sample of 16 out of 265 MPSs in the region was selected using a random sample of cases procedure in SPSS. Ten of these schools agreed to participate, eight of which were able to provide data on absence. Seven of the MPSs also provided data on pupil characteristics. All seven SSPEs in the region were asked to participate in the study, six of which agreed to participate and provide all data.

The eight participating MPSs had a total of 2216 pupils at the end of the school year. Three SSPEs did not supply the total number of pupils at the end of the year meaning that the total number of pupils in these three SSPEs at the end of the school year had to be estimated. This was done by taking the official total number of pupils in October 2015 and adding the average increase in pupils (9%) found in the other three SSPEs. This resulted in 24 additional pupils bringing the total estimated number of pupils in the six SSPEs to 1000.

The median age of pupils in the eight MPSs was 7.4 years. In the municipalities where the eight MPSs were located, 50% of pupils were boys. The median age of SSPE pupils was 9.4 years and 64% were boys [26, 31].

### Measures

The participating schools used a digital school absence registry to record each pupil’s absence daily. The school year 2015–2016 was analysed retrospectively. The school absence registry only contained those pupils recorded as absent, meaning that the number of pupils who were not absent in the chosen school year was not recorded. In order to determine the one-year prevalence of absence, the total number of pupils attending the school at the end of the school year was used.

Three types of authorised and two types of unauthorised absence were categorised: authorised comprised *sickness absence, doctor’s visits* and *other authorised absence* (such as family holidays or events requiring approval from the principal); unauthorised comprised *tardiness* and all *other unauthorised absence*, e.g. truancy.

When reported sick, pupils were labelled as sick either *occasionally* or *extensively* based on the definition of extensive sickness absence by Vanneste et al. of more than nine school days or more than four periods in a school year [29]. A period of absence is a single continuous span of time during which a pupil is absent. As soon as a pupil is registered as back in school, this period ends.

The frequency and duration (in half days) of all types of sickness absence, other authorised absence and other unauthorised absence were analysed. Additionally, the absence rate each of these absence types was determined, based on an estimated total of 180 possible school days in a school year. The absence rate is the ratio of absence days to possible school days. The duration of doctors’ visits and tardiness is not recorded by schools, therefore, only the frequency of these types of absence was analysed.

The month and year of birth, sex, years, and parental educational score of MPS pupils were collected from the school absence registry. Only the sex and the date of birth of pupils were available from SSPEs.

*Age* was calculated at the end of the school year based on the pupil’s month and year of birth.

For *years* MPS groups were made by combining lower years (Dutch school years one and two when pupils are normally four or five years-old), middle years (three, four and five) and senior years (six, seven and eight). Several schools had combination classes with different years in one class. These were allocated to the group of the highest year in each combination class.

The *parental educational score* was based on the parents’ highest educational achievement [32]. It was converted into a binary variable: category zero for parents with an education up to, or the equivalent of, prevocational education in the Netherlands, and category one for parents with a higher educational achievement than prevocational education.

### Analysis

Due to the variation in selection methods, data from MPSs and SSPEs were analysed separately. χ^2^ and Mann-Whitney U tests were used to analyse differences in occurrence of absence in MPSs and SSPEs. Univariate and multiple logistic regression analyses were used to determine the association between extensive sickness absence and (i) pupil characteristics (ii) other types of absence, and compared with occasional sickness absence.

The data were structured hierarchically, with pupils (first level) nested within schools (second level). In order to test if it was necessary to control for school effect in the analyses, the intra-class correlation coefficient (ICC) was determined [33, 34]. The ICCs for sickness absence ranged from .02 for MPSs to .07 for SSPEs, thus less than 8% of the variation in sickness absence in this sample was due to differences between schools, indicating that controlling for school effects was unnecessary.

## Results

### Study population

In MPSs 50% of pupils recorded in the absence registry were male, in SSPEs this was 64%. The mean age of pupils in SSPEs recorded in the absence registry was significantly higher than in MPSs (Median SSPEs: 9.64, MPSs: 7.95, Mann-Whitney test: U = 774.774.5, *p* < .001). Each MPS group (lower, middle and senior) contained approximately 33% of the pupils. A low parental educational score was found in 6% of MPS pupils. Concerning school size, the MPSs had an average of 277 pupils and the SSPEs an average of 167 pupils.

### Prevalence of school absenteeism

**Fig. 1:**
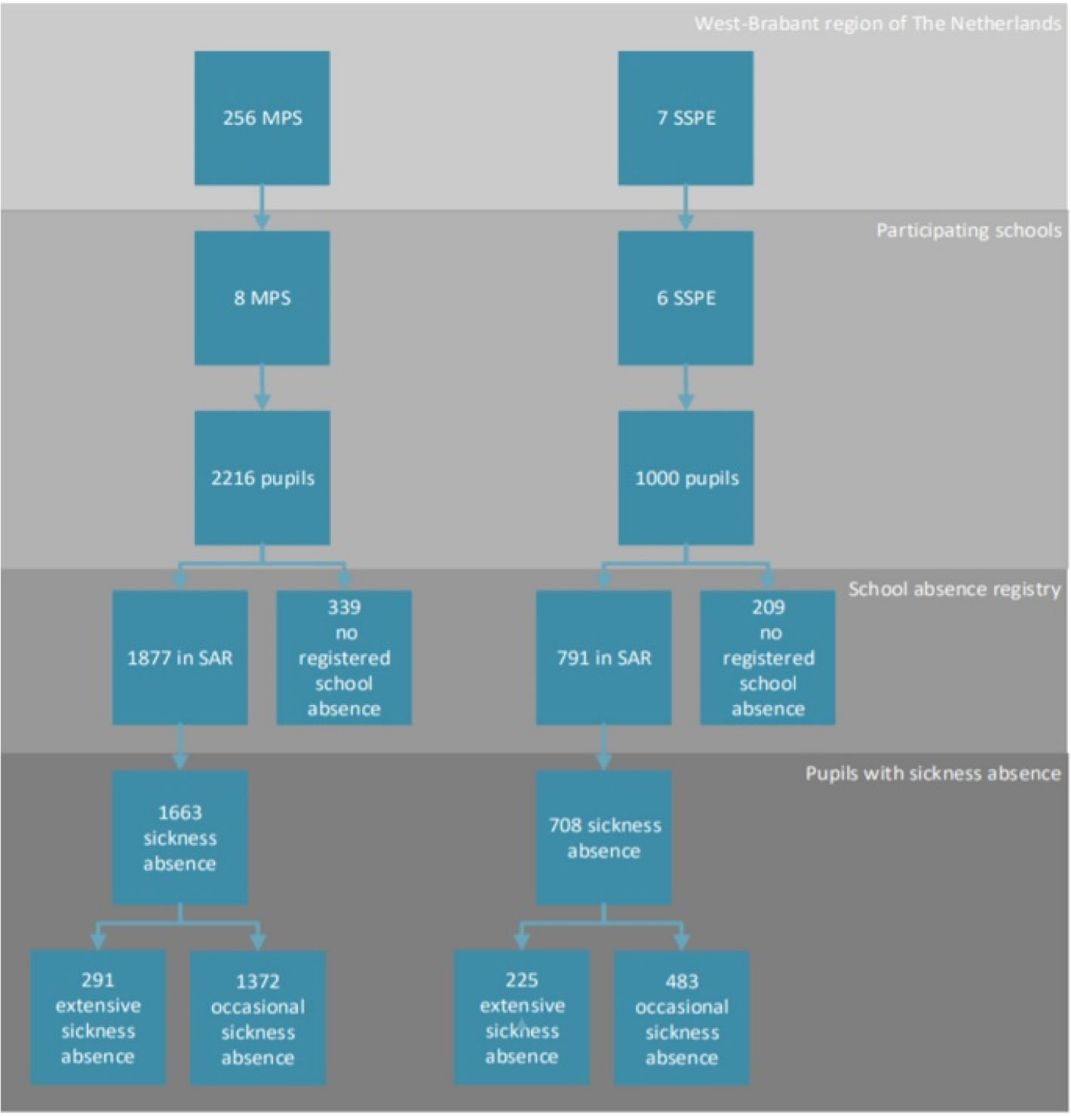
Overview of regular primary schools and pupil population in the region of West-Brabant

Of the 2216 pupils in MPSs, 85.70% (1877) were recorded as absent in the school year 2015–2016 (Fig. 1). In SSPEs 79.10% of the pupils (791) were recorded as absent. Sickness absence was the most frequently found (Table 1). In MPSs, 75.04% of pupils (1663) were reported sick at least once. Records showed that these pupils had a median of two periods of sickness (maximum of 31 periods), and three days (maximum of 45 days) in the school year. In SSPEs, 70.80% of pupils (708) were reported sick at least once during the year, with a median of three periods and four days of sickness absence (maximum: 28 periods and 80 days). Extensive sickness absence was recorded in 13.13% of MPS pupils and 22.50% of SSPE pupils. Unauthorised absence was the least prevalent type of absence. Other than tardiness, unauthorised absence was recorded in 1.81% of MPS pupils and 8.00% of SSPE pupils. The frequency and duration of types of school absence are shown in Table 2.

**Table 1:** Types of school absence in primary education in the school year 2015/2016

Type of school absence	MPS: Number of pupils (%)	SSPE: Number of pupils (%)
	***N = 2216***	***N = 1000***
Sickness absence	1663 (75.0) *	708 (70.8)
Occasional sickness absence	1372 (61.9) †	483 (48.3)
Extensive sickness absence	291 (13.1) †	225 (22.5)
One or more long period of sickness absence (> 9 days and < 4 periods)	42 (1.9) †	19 (1.9)
High frequency of sickness absence (> 4 periods and < 9 days)	129 (5.8) †	79 (7.9)
Both long periods of sickness absence and high sickness absence frequency (> 9 days and > 4 periods)	120 (5.4) †	127 (12.7)
Doctor’s visits	574 (25.9) **	314 (31.4)
Other authorised absence	539 (24.3) †	337 (33.7)
Tardiness	219 (9.9) **	135 (13.5)
Other unauthorised absence	40 (1.8) †	80 (8.0)

MPS: Mainstream primary schools

SSPE: Special schools for primary education

* *p* < 0.05, ** *p* < 0.01, † *p* < 0.001 at 95% confidence interval between MPS and SSPE

**Table 2:** Frequency and duration of school absence among pupils in primary education in the school year 2015/2016

		MPS (total N pupils:2216)			SSPE (total N pupils:1000)		
		Mean (SD)	Median	Range	Mean (SD)	Median	Range
Sickness absence	Duration (days)	4.30 (4.44)	3†	0.5–63	6.24 (6.94)	4	0.5–80
	Frequency	2.73 (2.36)	2†	1–31	3.74 (3.26)	3	1–28
Doctor’s visits	Frequency	2.18 (3.13)	1†	1–35	2.39 (1.87)	2	1–12
Other authorised absence	Duration (days)	2.52 (4.15)	1†	0.5–62.5	1.65 (1.60)	1	0.5–12
	Frequency	1.85 (2.86)	1†	1–56	1.73 (1.99)	1	1–24
Tardiness	Frequency	4.91 (8.44)	2†	1–66	4.55 (6.59)	2	1–43
Unauthorised absence	Duration (days)	1.05 (0.98)	1†	0.5–5.5	1.93 (2.56)	1	0.5–13.5
	Frequency	1.15 (0.43)	1†	1–3	1.86 (2.36)	1	1–14

MPS: Mainstream primary schools

SSPE: Special schools for primary education

† Significant difference between MPS and SSPE at 95% confidence interval *p* < 0.001

Focusing on absence rates, the total absence rate in MPSs was 2.15% and the sickness absence rate was 1.80%. The rate of other authorised absence was 0.34% and that of other unauthorised absence was 0.01%. In SSPEs the absence rates were 2.85, 2.45, 0.31 and 0.09%, respectively.

When comparing sickness absence in SSPEs with MPSs, SSPE pupils were reported sick significantly more often (median SSPEs: 3, MPSs: 2, Mann-Whitney test: U = 696.175,500, *p* < .001), and for longer (median SSPEs: 4, MPSs: 3, Mann-Whitney test: U = 701.361,500, *p* < .001) than MPS pupils. The rate of extensive sickness absence was significantly higher in SSPEs than in MPSs (χ^2^ (1) = 59.483, *p* < =.001).

### Factors associated with extensive sickness absence

Table 3 shows the results of both univariate and multivariate logistic regression analyses. Only the variables that were statistically significant in the univariate analysis were included in the multivariate logistic regression analysis. Multivariate analysis of extensive sickness absence among MPS pupils showed a statistically significant relationship with lower age, lower parental educational score, doctor’s visits, and other unauthorised absence, when compared with pupils with occasional sickness absence. Among SSPE pupils all other types of school absence showed a statistically significant relationship with extensive absence. This indicates that in addition to extensive sickness absence, these pupils are also more often reported absent for other reasons.

**Table 3:** Logistic regression analysis of factors associated with extensive sickness absence (versus occasional sickness absence)

	MPS					SSPE				
	Number of Pupils	Univariate		Multivariate		Number of Pupils	Univariate		Multivariate	
		Exp(B)	CI	Exp(B)	CI		Exp(B)	CI	Exp(B)	CI
Sex ^◊^	1525	0.98	0.75–1.28	–	–	705	0.98	0.70–1.37	–	–
Age	1513	0.93*	0.89–0.99	0.92*	0.87–0097	589	1.01	0.93–1.10	–	–
Low parental education level	1524	2.00*	1.25–3.18	1.66*	1.01–2.72	–	–	–	–	–
Doctor’s visits ▪	1530	2.14**	1.63–2.83	2.26**	1.69–3.01	708	1.63**	1.18–2.25	1.48*	1.05–2.08
Other authorised absence ▪	1530	1.42*	1.06–1.89	1.21	0.90–1.64	708	1.94**	1.41–2.68	1.90**	1.36–2.65
Tardiness ▪	1530	1.22	0.80–1.85	–	–	708	2.67**	1.79–3.99	2.35**	1.54–3.59
Other unauthorised absence ▪	1530	5.06**	2.38–10.74	4.58**	2.08–10.11	708	3.22**	1.98–5.23	2.75**	1.66–4.54

MPS: Mainstream primary schools, SSPE: Special schools for primary education, CI: Confidence Interval

◊ reference: male

▪ having any of this type of absence at least once during the academic year 2015/2016. Reference: having none of this type of absence

* *P* < 0.05, **P < 0.01, 95% confidence interval

## Discussion

This study was performed in eight MPSs with 2216 pupils and six SSPEs with 1000 pupils in order to gain insight into the prevalence of school absence and the relationship between extensive sickness absence and pupil characteristics and other types of school absence.

### Prevalence of school absenteeism

Most pupils, 85% in MPSs and 79% in SSPEs, were absent at least once during the school year, with total school absence rates of 2.1 and 2.9%, respectively. The most common type of absence from school in primary schools was absence due to sickness: 75% of MPS pupils and 71% of SSPE pupils were reported sick at least once in the school year, with sickness absence rates of 1.8 and 2.5%, respectively. While comparable research is limited, reports from Scotland and England were available and show similar figures with total school absence rates of 5.0% in Scotland and 4.0% in England, and sickness absence rates of 2.9 and 2.4%, respectively [35, 36].

In the current study, unauthorised absence occurred rarely in MPSs (1.8% of pupils) and more frequently in SSPEs (8.0%). However, even 8% in SSPE seems low when considering that research often focuses on unauthorised absence [2, 18]. In Dutch, Scottish and English primary schools, sickness absence is clearly the most prevalent type of absence. Although comparison of prevalence between studies is difficult as findings may be influenced by differing selection methods, type of school and the way absence is measured. In the USA Cook et al. developed a primary school absence programme and found that only 47% of all absences were authorised [24]. Sickness absence was not specifically mentioned. The high prevalence of sickness absence and low prevalence of unauthorised absence found in the current study might be explained by the fact that it is easy to report a child as sick in the Netherlands, and it may be more convenient for a parent to report their child as sick than explain unauthorised absence to the authorities.

### Extensive sickness absence

This study found that extensive sickness absence occurred frequently: 13% of MPS pupils and 22.5% of SSPE pupils were reported sick for more than four periods or more than nine days.

The current study showed that extensive sickness absence in MPSs occurred more often in younger pupils, and where parents had a lower educational level. A young age has previously been described as related to chronic school absence [6]. The relationship between extensive sickness absence and age may be due to childhood diseases in younger children [6, 37], or may be related to the start of mandatory attendance. Dutch primary education starts at four years-old and lasts eight years [38], however, the first year is not mandatory. The relationship found between a lower parental educational score and extensive sickness absence is in line with other studies that also found parental education and lower socio-economic status were associated with more absence from school [10, 18, 25].

In SSPEs, extensive sickness absence is associated with all other types of school absence, irrespective of pupil age. In MPSs extensive sickness absence is associated with more doctor’s visits and unauthorised absence. In addition to days missed due to extensive sickness absence, pupils miss even more days in school for other reasons, when compared with those who are only reported sick occasionally.

### MPS vs. SSPE

Compared with MPS, SSPE pupils were slightly less often reported as absent (85% vs 79%, respectively) or sick (79% vs 71%). However, regarding the subsamples of sickness absence, the sickness absence frequency and duration was higher in SSPEs than in MPSs. The reasons for attending SSPE, e.g. behavioural, learning and parental factors, have previously been described as influencing school absence [10], and thus the differences in the frequency and duration of sickness absence between MPS and SSPE might be explained by these factors. Whether behavioural, learning or parental factors cause sick reporting either directly or through increased vulnerability to illness, is unknown.

### Strengths and limitations

As the age and sex distribution of pupils in the absence registry (MPSs: 7.95 years-old, 50% boys, SSPEs: 9.64 years-old, 64% boys) were all similar to their national equivalent (MPS: 7.87 years-old, 51% boys, SSPE: 9.57 years-old and 67% boys) [26, 31], the results of this study appear to be generalisable to those in other areas in the Netherlands.

To determine the occurrence of school absence, the total number of pupils at the end of the school year were used, rather than the total at the start. As more pupils enrol than leave during the school year, the totals at the start of the school year would have given an overestimation of absence. However, as late enrolees have less opportunity to be absent, using the end of the school year means that school absence might be even higher than found in this study.

In this sample, the average school size (MPS: 277 pupils and SSPE:167 pupils) was moderately larger than the national average (MPS: 224 pupils and SSPE: 122 pupils), and prevalence of MPS pupils with a low parental educational score (6%) was lower than the national average of 9% [32]. As a larger school size has previously been shown to be related to more school absence [9], and a low educational score was associated with more extensive sickness absence, the national prevalence of extensive sickness absence may well be even higher than found in this study.

Schools that did not agree to participate in this study stated time constraints and once, low prevalence of sickness absence among pupils as the main reason. It is unknown whether the prevalence of school absence in these schools is actually different.

### Extensive sickness absence

Using a threshold for extensive sickness absence creates the opportunity to compare groups. The design of the threshold used in this study was based on interviews in schools and theorised that the pupils most at risk of negative consequences were those with sickness absence 1SD above the average sickness absence frequency or duration (as reported in a pilot study) [29]. As the groups selected in the current study had extensive sickness absence and missed additional days due to other types of absence, it appears that a vulnerable group was selected. Whether or not this threshold selects the most vulnerable pupils has not been tested.

### Absence registration

The absence data were recorded daily by school employees and were collected retrospectively, thus minimising recall and information bias. Simultaneously, using retrospective analyses left no opportunity to improve the accuracy of recording absence. According to participating schools, not all teachers recorded every absence. Tardiness in particular might be subject to underreporting as not all schools expect punctuality. Previous studies have reported on the variations in recording practices [24, 35, 39], therefore it is not unlikely that this may also have caused underreporting in the current data. The size of the current sample, i.e. 14 schools with 3216 pupils, minimises the effect of individual recording mistakes.

### Recommendations for further research

Most absence in the participating Dutch primary schools was because pupils were reported sick, which is similar to reports from Scotland and England [35, 36]. Traditionally, the focus of research into school absence has been on unauthorised absence, possibly because of a lower prevalence of sickness absence in other countries such as the USA. Another explanation could be that, as its cause seems medical, sickness absence is seen as inevitable. However, this study suggests that learning, behavioural and parental factors may also play a role. More research is therefore needed to determine the prevalence of sickness absence in other countries and to determine the factors that influence sickness absence. Country-specific approaches to defining, recording and addressing school absence should be taken into account when examining this topic.

The threshold used for extensive sickness absence should be further examined to determine if those pupils who are most vulnerable to adverse outcomes can be selected using these criteria, and if these criteria should be adjusted when used in other countries.

## Conclusions

This study shows that in Dutch primary education school absenteeism is most often due to children being reported sick. Moreover, extensive sickness absence is common (13.1% in MPSs and 22.5% in SSPEs), and occurred more often in SSPE pupils than in MPS pupils. In MPSs, younger pupils and pupils with parents with a lower educational level appeared most at risk of extensive sickness absence. Additionally, in comparison with pupils with occasional sickness absence, pupils with extensive sickness absence were absent on more days for reasons other than sickness. Thus, these pupils miss even more days of school, likely increasing their disadvantage by missing lessons and contact with their peers. Combined with the high prevalence of extensive sickness absence found in this study, this is reason to worry. To prevent adverse effects on children’s development it is of utmost importance that public health research, policy and practice address sickness absence among primary school pupils.

## Data Availability

The dataset generated and analysed during the current study is not publicly available due to data protection rules but can be made available from the corresponding author on reasonable request.

## Abbreviations

ICC: intra-class correlation coefficient
MPS: mainstream primary school
SD: standard deviation
SSPE: special school for primary education
